# Optimization of the Nutritional Parameters for Enhanced Production of *B. subtilis* SPB1 Biosurfactant in Submerged Culture Using Response Surface Methodology

**DOI:** 10.1155/2012/795430

**Published:** 2012-05-07

**Authors:** Ines Mnif, Semia Chaabouni-Ellouze, Dhouha Ghribi

**Affiliations:** ^1^Unité Enzymes et Bioconversion, Ecole Nationale d'Ingénieurs de Sfax, BP W 3038, Sfax, Tunisia; ^2^Higher Institute of Biotechnology of Sfax, BP 261, 3000, Sfax, Tunisia

## Abstract

Nutritional requirements can contribute considerably to the production cost and the bioprocess economics. Media optimisation using response surface methodology is one of the used methods to ameliorate the bioprocess economics. In the present study, biosurfactant production by *Bacillus subtilis* SPB1 was effectively enhanced by response surface methodology. A Plackett-Burman-based statistical screening procedure was adopted to determine the most important factor affecting lipopeptide production. Eleven variables are screened and results show that glucose, K_2_HPO_4_, and urea concentrations influence the most biosurfactant production. A Central Composite Design was conducted to optimize the three selected factors. Statistical analyses of the data of model fitting were done by using NemrodW. Results show a maximum predicted biosurfactant concentration of 2.93 (±0.32) g/L when using 15 g/L glucose, 6 g/L urea, and 1 g/L K_2_HPO_4_. The predicted value is approximately 1.65 much higher than the original production determined by the conventional one-factor-at-a-time optimization method.

## 1. Introduction

Biosurfactants are surface active compounds with widely varied structures. They correspond to amphiphilic molecules with a hydrophilic (amino acids, peptides, anionic or cationic, di-or polysaccharides) and a hydrophobic (saturated or unsaturated fatty acid) moieties, which are synthesised by a wide spectrum of microorganisms [1]. They are categorized mainly by their chemical composition and their microbial origin. Consequently, the major classes of biosurfactants include glycolipids, lipopeptides and lipoproteins, phospholipids and fatty acids, polymeric surfactants, and particulate surfactants [1]. Predominantly, biosurfactants are synthetised by a variety of microorganisms during growth on water-immiscible substrates [1]. They have several properties, increasing the surface and interfacial tension between surface and interface, respectively, with very low critical micelle concentration, none toxicity and highly biodegradability and tolerating extreme conditions such as high temperature value, extreme pH, and high salinity [2]. Furthermore, biosurfactants offer numerous biological activities for increasing commercial importance. For this reasons, in the past few decades, they showed great economic interest, specifically, in environmental field as a biocontrol agent and for their insecticide activity, in bioremediation for their role in hydrocarbon contaminant biodegradation and sequestering; in chemical industry, food processing, food additives, cosmetic, and pharmaceuticals field for their emulsifying, foaming, dispersant, and antiadhesive activities in medicine for their antimicrobial, antitumoral, antiviral, and anti-inflammatory activities [1, 3]. Nevertheless, the high cost of fermentation and downstream processing limit the large-scale production of biosurfactants and their use. Thus, many scientists focus in enhancing the microbial production of surfactants. To improve yield production, many methods are possible like media optimisation, agro-industrial waste fermentation, and strain improvement by mutagenesis or recombinant strains [2]. One of the methods of achieving the above objective is the selection of appropriate media components and optimal culture conditions for maximum biosurfactant productivity. In fact, the nature of the carbon substrate, the concentration of N, P, Na, Mg, Fe, Zn, and Mn ions in the medium, and operational conditions, such as pH, temperature, agitation, and aeration have been shown to influence enormously the nature and quantity of the biosurfactant produced by several strains [1, 2, 4–6]. In the present work, we adopted a planning experimental methodology to enhance the production of lipopeptide biosurfactant by *B. subtilis *SPB1. These include a first screening by Plackett-Burman design and an optimization by a Central Composite Design.

## 2. Materials and Methods

### 2.1. Microorganism Strain and Biosurfactant Production

*Bacillus subtilis* SPB1 (HQ392822) was a wild-type strain isolated from Tunisian soil [7]. It was characterized in our laboratory as a producer of a lipopeptide biosurfactant with highly emulsifying activity. Culture conditions were carried out as described by Ghribi and Chaabouni [7]. The production medium was composed of glucose, urea, ammonium chloride, sodium chloride, and other salts (Table 1). The pH was adjusted to 7 prior to sterilization. All experiments were performed in triplicate. At the end of the cultivation, the culture was centrifuged at 10000 rpm and 4°C for 20 min to remove bacterial cells. The supernatant free cells served for biosurfactant extraction.

### 2.2. Preparation of the Crude Extract

The supernatant was acidified using 6 N HCl under pH 2, incubated at 4°C overnight, and centrifuged for 20 min at 4°C at 10.000 rpm to collect the grey pellets formed. The pellet formed was washed three times with acid water (pH 2) to collect the crude lipopeptide preparation. Pellets corresponding to the crude surfactant were weighted for quantification after desiccation at 105°C for 24 h. The values presented are the average of the results of three determinations of three separate experiments for each cultural condition.

### 2.3. Experimental Design and Statistical Analysis for Determination of the Critical Medium Components

#### 2.3.1. Identification of Important Nutrient Components: Plackett-Burman Experimental Design

To find out the important medium components, a Plackett-Burman design was applied (Table 2). This design is a fractional plan. It allows the investigation of up to N-1 variables with N experiments and assumes that there are no interactions between the different media components [8]. For this study, 11 components were selected to evaluate their effect on biosurfactant production. A total of 17 experiments were conducted including 12 experiments of the Hadamard matrix (Run N°1 to 12) and 5 experiments in the domain centre (Run N°13 to 17) as shown in Table 2. Each variable was assessed at three coded levels (−1, 0 and +1). The various media components included in Plackett-Burman experiments and their corresponding higher, medium, and lower concentration levels are presented in Table 1.

A linear approach is considered to be sufficient for screening

(1) $$\begin{array}{c} Y=\beta_{0}+\beta_{i}f_{i}\;\left(i=\;1\cdots k\right), \end{array}$$

where *Y* is is the response (biosurfactant production yield g/L), *β*_*i*_ are the regression coefficients, and *f*_*i*_ is the level of the independent variable. The contrast coefficient, noted *b*, was calculated as the difference between the average of measurements made at the high (+) and the low (−) levels of the factors. This coefficient notifies the main effect of the studied factor [8]. The significance of each variable was determined via a Student's *t* test by the statistical software package SPSS (version 17). The five replicates at the center point of the design permitted to estimate the variability of the experimental results.

#### 2.3.2. Optimization of Screened Components by Response Surface Methodology: Central Composite Design Experiments

In order to determine the optimum values of the most influent factors, to obtain an empirical model of the process and to improve biosurfactant production, we adopted a central composite design. It consists of a complete 2^*k*^ factorial design, where *k* is the number of the test variables and is equal to 3, five replications of the center points to estimate the experimental error and have a satisfactory orthogonality for the coefficients estimation (all factors at level 0), six star points (2 axis points on the axis of each variable at a distance of *α* ( = 2^*k*/4^, = 1,682 for *k* = 3), whereas the other two factors are at level 0 and four tests points. Hence, the total number of design points is *N* = 2^*k*^ + 2*k* + *n*_0_ + 4 = 23 experiments. The central composite design along with the experimental and predicted values of biosurfactant production was showed in Table 3. The response values ($\hat{y}$) used in each trial was the average of the duplicates.

#### 2.3.3. Statistical Analysis and Modelling

The data obtained from the central composite design with regards to biosurfactant production were subjected to analysis of variance (ANOVA) to check for errors and the significance of each parameter. Biosurfactant production yield was taken as response ($\hat{Y}$). The data were then subjected to a multiple regression analysis to obtain an empirical model that could relate the response measured to the independent variables. The behaviour of the system was explained by the following quadratic equation:

(2) $$\begin{array}{cc} \hat{Y} & =b_{0}+b_{1}X_{1}+b_{2}X_{2}+b_{3}X_{3}+b_{11}X_{1}^{2}+b_{22}X_{2}^{2}\;\\ & \;+b_{33}X_{3}^{2}+b_{12}X_{1}X_{2}+b_{13}X_{1}X_{3}+b_{23}X_{2}X_{3}, \end{array}\;\;$$

where $\hat{Y}$ refers to the predicted response, *X*_1_, *X*_2_, *X*_3_ to the independent coded variables, *b*_0_ to the offset term, *b*_1_, *b*_2_, *b*_3_ to the linear effects, *b*_11_, *b*_22_, *b*_33_ to the squared effects, and *b*_12_, *b*_23_, *b*_13_ to the interaction terms.

The statistical software package, (Nemrod-W by LPRAI Marseilles, France) [9] was used to conduct a regression analysis on the experimental data and to plot the response surface graphs. The statistical significance of the model was determined by the application of Fisher's *F* test [10]. The two-dimensional graphical representation of the system behaviour, called the isoresponse contour plot, was used to describe the individual and cumulative effects of the variables as well as the possible correlations that existed between them.

## 3. Results

### 3.1. Identification of Important Nutrient Components: Plackett-Burman Experimental Design

In order to determine the critical media components affecting biosurfactant production by *Bacillus subtilis* SPB1, the Plackett-Burman experiments were conducted. Table 1 represents the nine independent variables and their respective high and low values used in the statistical screening study. Table 2 represents the Plackett-Burman experimental design for 12 trials at two levels of concentration for each variable and the 5 trials at centre point of the variables along with responses (biosurfactant yield). The 17 experiments were carried out in triplicate and the averages of results were presented. The data were analyzed using the statistical software package SPSS. These data permitted the estimations of the model coefficients, b_i_, using multilinear regression.

To remember, the Plackett-Burman design assumes that there are no interactions between the different factors. Hence, a linear approach is considered to be sufficient for screening. The effects of various nutritional factors on biosurfactant production based on the observations of Plackett-Burman design experiments were shown in Table 3. Results showed that the main parameters affecting the production of the lipopeptide biosurfactant were determined as glucose, urea, and K_2_HPO_4_ with contrast coefficient of 0.551, 0.415, and 0.520, respectively, and very low *P* values of less than 0.01 (0.0006; 0.0024, and 0.0009, resp.). They are highly significant at very high confidence levels (>99%). Therefore, they were retained for further optimization using a central composite design. Also, FeSO_4_ and CaCl_2_ concentrations affect significantly the production yield but according to their coefficient values (−0.216 and 0.240 resp.), they affect negatively the response, so they were retained at their low levels in the continuation of the work.

### 3.2. Central Composite Design Experiments

#### 3.2.1. Analysis of Variance and Validation of the Model

The three parameters identified as having important effects on biosurfactant production by the screening experiments (glucose, urea, and K_2_HPO_4_) were optimized using Central Composite Design. The experimental and the predicted responses were presented in Table 4. Results were the average of three independent essays. The levels of the other parameters were fixed at their low levels for the experiments.

The experimental results were modeled with a second-order polynomial equation to explain the dependence of biosurfactant production on the different factors:

(3) $$\begin{array}{cc} Y & =2.877-0.253X_{1}-0.056X_{2}-0.130X_{3}-0.240X_{1}^{2}\\ & \;+0.008X_{2}^{2}-0.204X_{3}^{2}+0.100X_{1}X_{2}+0.025X_{2}X_{3}, \end{array}\;$$

where *Y* was the estimated biosurfactant production and *X*_1_, *X*_2_, and *X*_3_ were the coded values for glucose, K_2_HPO_4_, and urea concentrations, respectively.

Statistical analysis of results was performed to determine the significant differences. The significance of each coefficient was determined by Students's *t*-test. The Student t distribution and the corresponding *P* values, along with the parameter estimate, were given in Table 5. As clear, five out of the ten variables included in this study were found to be statistically highly significant in the biosurfactant production process. By considering only the significant factors, biosurfactant production could be predicted by the following equation:

(4) $$\begin{array}{c} Y=2.877-0.253X_{1}-0.130X_{3}-0.240X_{1}^{2}-0.204X_{3}^{2}. \end{array}$$

According to this equation it is well described that biosurfactant yield can be estimated as a function of the linear effect of glucose concentration, the linear effect of urea concentration, and the squared effect of glucose. 

The statistical significance of the model was checked by *F*-test and the results were presented in Table 6. ANOVA analysis for biosurfactant production showed that the regression model was significant and the lack of fit was insignificant (Table 6). The fit of the models was evaluated by the determination of coefficient *R* ^2^. The regression equations obtained indicated the *R* ^2^ values of 0.915 suggesting an adequate adjustment of the quadratic model to the experimental data and indicating that the model could explain 91.50% of the variability in the response. The closer the values of *R* ^2^ to 1, the better the model would explain the variability between the experimental and the model predicted values [11].

#### 3.2.2. Graphical Interpretation of the Response Surface Model: Optimization of the Significant Nutrient Components

**Figure 1:**
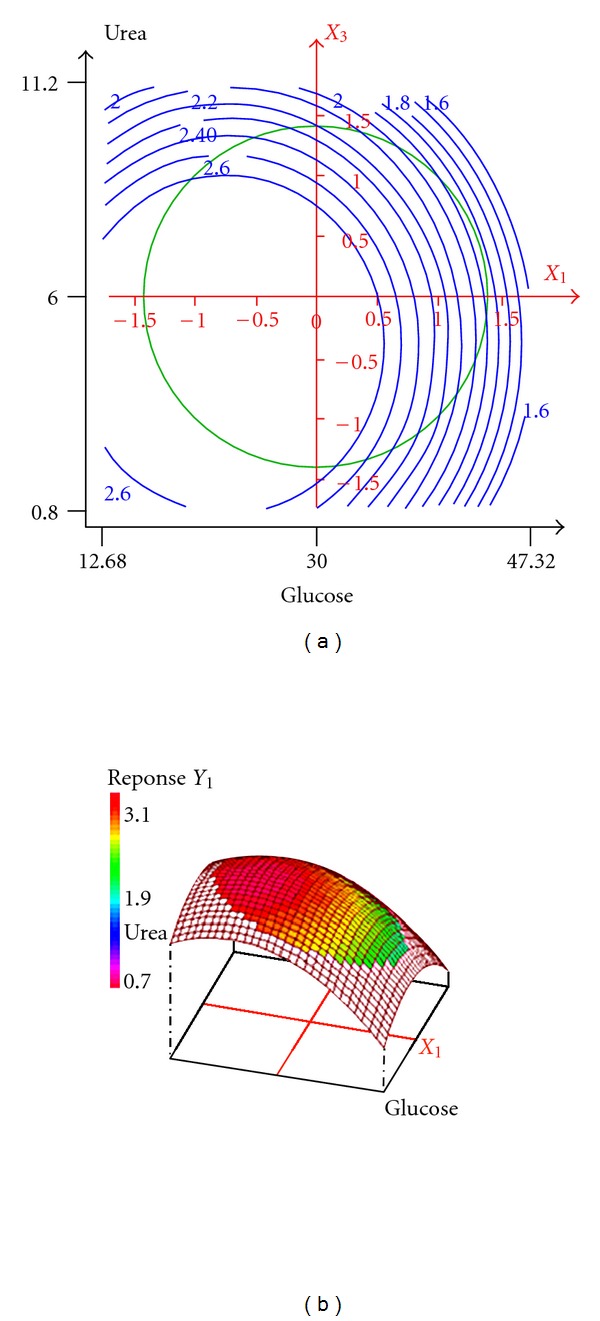
Effect of glucose and urea concentration on biosurfactant production yield: response surface plot (a) and its contour plot (b) of interaction between glucose concentration and urea concentration with K_2_HPO_4_ concentration kept at 1 g/L.

The effect of the interaction of various nutritional parameters on biosurfactant production by *B. subtilis *was investigated by plotting the response surface curves against any two independent variables while keeping the third independent variable at constant level. The response surface plots and their respective contour plots for the predicted response *Y* (biosurfactant production yield), based on the second-order model are shown in Figure 1. They provided information about the interaction between two parameters and allowed an easy interpretation of the results and prediction of the optimal values. According to Table 5, the linear, quadratic effect of the second parameters and the interaction between *X*_2_ and *X*_1_ and between *X*_2_ and *X*_3_ are insignificant. So, we have fixed the concentration of K_2_HPO_4_ 1 g/L. So, as described in (4), the response was represented as function of the interaction between glucose and urea concentration (Figure 1). This interaction was investigated by plotting the 3D response surfaces with the vertical axis representing biosurfactant production yield and two horizontal axes representing the coded levels of two explanatory factors. The optimal values for the variables were obtained by moving along the major and minor axis of the contour. In fact, when biosurfactant production was observed as a response to the interaction of glucose and urea concentrations as variables and K_2_HPO_4_ concentration at low point, it was observed that there was an enhancement in biosurfactant production at lower glucose concentration and middle urea concentration (Figure 1). 

As a result, based on the 3D plots, the optimal concentration values for *X*_1_, *X*_2_, and *X*_3_ (glucose, K_2_HPO_4_, and urea) were identified as 15, 1, and 7.5 g/L, respectively. The corresponding experiment was carried out in five replicates and the average value was calculated. The biosurfactant production was about 3.1 g/L while the predicted value was 2.93(±0.32) g/L. This confirms the closeness of the model to the experimental results.

## 4. Discussion

Production economy is the major interest in secondary metabolites production, as in the case with most biotechnological processes. Often, the amount and type of fermentative media components can contribute considerably to the production cost [2]. One possibility explored extensively is the application of experimental planning methodology to enhance biosurfactant production through optimization of nutritional requirements. Liquid fermentation with the use of simple substrates is almost the more utilized to produce lipopeptide biosurfactant. Nutritional parameters affect highly the production yield and cost [1, 2]. Several carbon sources like carbohydrates, starchy substrates, vegetable oils, and hydrocarbon are utilized to produce lipopeptide by *Bacillus* strains [12–14]. According to Ghribi and Chaabouni (2011) [7], *B. subtilis *SPB1 was able to use many carbon sources like glucose, sucrose, starch, and glycerol to produce lipopeptide but the use of glucose as carbon source seems to be more interesting. Different other media components, such as nitrogen sources, salts elements like iron and manganese are reported to affect the process of biosurfactant production and the final quality and quantity [2, 15, 16]. Therefore, in order to reach overproduction of lipopeptide biosurfactants by *B. subtilis* SPB1, nutritional requirements were studied using the experimental design methodology. According to previous reports and studies, eleven nutritional factors including glucose, urea, ammonium sulfate, sodium chloride concentrations, and several salts concentrations were selected as the key factors affecting the production yield in the present investigation. They were shown to influence considerably biosurfactant production in many previous reports [15–20]. In the first step, a Plackett-Burman design was conducted to screen the most influent parameters on the production yield. Among the 11 medium component tested, glucose, K_2_HPO_4_, and urea concentrations were found the most important parameters influencing biosurfactant production. Results were in accordance to those reported by Abushady et al., 2005 [19], Sivapathasekaran et al., 2010 [21], and Mukherjee et al., 2008 [22]. In the second step, a central composite design was carried out to determine the optimal levels of the three selected variables. To remember, the classical method of optimization, by a conventional “one-at-a-time-approach” is not only cumbersome and time consuming, but also has the limitations of ignoring the importance of interaction of various parameters and can lead to wrong results. Response surface methodology permits to study the interaction between the different parameters and to determine their optimal levels. A high degree of similarity was observed between the predicted and experimental values that reflected the accuracy and applicability of response surface methodology to optimise the process for biosurfactant production. A maximum production yield of about 3.1 g/L lipopeptide biosurfactant was achieved when using glucose, urea, and K_2_HPO_4_ at concentrations of 15, 7.5, and 1 g/L, respectively and keeping the other parameters at their minimum values suggesting the necessity of salts elements for biosurfactant production. The elimination of these factors may cause a disruption of the response. In fact, carbon and nitrogen sources presented a determinant effect on metabolite production [23, 24]. The nature and the quantity of the carbon source were found as the most important factors that would affect biosurfactant production [5, 25, 26]. Among all the tested substrates, the use of glucose as carbon source to produce biosurfactants seems to be most interesting [21, 27, 28]. Glucose quantity requirements by *B. subtilis* SPB1 was much lower than those described in other previous reports [21, 22, 27]. Urea [21] and K_2_HPO_4_ [18, 22, 27] were also reported to improve lipopeptide production. Based on the optimization experiments, it can be concluded that the biosurfactant production by *B. subtilis* SPB1 was enhanced to 1.65-fold over the original production determined by the conventional one-factor-at-a-time optimization method [7]. Also, this permits an economic gain through the reduction of glucose concentration and the elimination of kerosene. In fact, according to the previous study, we can reach a production yield of 1.74 g/L when using 40 g/L glucose and 2% of kerosene [7].

## 5. Conclusion

In order to enhance biosurfactant production by *B. subtilis* SPB1, nutritional requirements were studied using response surface methodology. A statistical screening procedure using a Plackett-Burman design was adopted to select the main factors affecting lipopeptide production. Estimation and statistical analysis of coefficient in Plackett Burman design experiments demonstrate that glucose, urea, and K_2_HPO_4_ affect the most biosurfactant production. Optimization of these three selected variables while keeping the rest of the factors at their low levels through a Central Composite Design shows a maximum predicted biosurfactant concentration of 2.93(±0.32) g/L when using 15 g/L glucose, 7.5 g/L urea and 1 g/L K_2_HPO_4_. The production yield is approximately 1.65 much higher than the original production. This suggests the effectiveness of statistical tools in bioprocess optimization with a large gain of cost and time. In fact, response surface methodology was demonstrated in many literature studies as an efficient tool to optimize metabolites production by several strains.

## Figures and Tables

**Table 1:** The various media components included in Plackett-Burman experiments and their corresponding higher, medium, and lower concentration levels.

Variables code	Media constituents	Units	High level (+1)	Medium level (0)	Low level (−1)
*F*_1_	Glucose	g/L	40	25	10
*F*_2_	Urea	g/L	5	3	1
*F*_3_	Ammonium sulfate	g/L	5	3	1
*F*_4_	NaCl	g/L	5	2.75	0.5
*F*_5_	MgSO_4_	g/L	2	1.1	0.2
*F*_6_	KH_2_PO_4_	g/L	2	1.25	0.5
*F*_7_	K_2_HPO_4_	g/L	2	1.25	0.5
*F*_8_	MnSO_4_	g/L	0.01	0.0055	0.001
*F*_9_	FeSO_4_	g/L	0.01	0.0055	0.001
*F*_10_	ZnSO_4_	g/L	0.01	0.0055	0.001
*F*_11_	CaCl_2_	g/L	0.01	0.0055	0.001

**Table 2:** Plackett-Burman experimental design for 11 variables and the corresponding responses in g/L.

Factors (coded)												
Exp N°	*F*_1_	*F*_2_	*F*_3_	*F*_4_	*F*_5_	*F*_6_	*F*_7_	*F*_8_	*F*_9_	*F*_10_	*F*_11_	Biosurfactant yield (g/ L)
1	1	1	−1	1	1	1	−1	−1	−1	1	−1	2.06
2	−1	1	1	−1	1	1	1	−1	−1	−1	1	1.61
3	1	−1	1	1	−1	1	1	1	−1	−1	−1	2.22
4	−1	1	−1	1	1	−1	1	1	1	−1	−1	1.8
5	−1	−1	1	−1	1	1	−1	1	1	1	−1	0.7
6	−1	−1	−1	1	−1	1	1	−1	1	1	1	0.7
7	1	−1	−1	−1	1	−1	1	1	−1	1	1	1.8
8	1	1	−1	−1	−1	1	−1	1	1	−1	1	1.7
9	1	1	1	−1	−1	−1	1	−1	1	1	−1	1.9
10	−1	1	1	1	−1	−1	−1	1	−1	1	1	0.9
11	1	−1	1	1	1	−1	−1	−1	1	−1	1	0.8
12	−1	−1	−1	−1	−1	−1	−1	−1	−1	−1	−1	0.3
13	0	0	0	0	0	0	0	0	0	0	0	1.52
14	0	0	0	0	0	0	0	0	0	0	0	1.49
15	0	0	0	0	0	0	0	0	0	0	0	1.59
16	0	0	0	0	0	0	0	0	0	0	0	1.54
17	0	0	0	0	0	0	0	0	0	0	0	1.48

**Table 3:** Estimated effect, regression coefficient, and corresponding *t*, *P* values and confidence level of each variable described for biosurfactant production in Plackett Burman design experiments.

Noun	Coefficient	F Inflation	SE	*t*.exp	*P* value	Confidence level (%)	Signification
*b*_0_	1.418		0.209	−1.307	0.2480	75.2	NS
*b*_1_	0.551	1.00	0.003	7.447	0.0006	99.94	***
*b*_2_	0.415	1.00	0.023	5.611	0.0024	99.76	**
*b*_3_	0.013	1.00	0.023	0.186	0.8597	14.03	NS
*b*_4_	0.107	1.00	0.020	1.446	0.2077	79.23	NS
*b*_5_	0.184	1.00	0.051	2.490	0.0551	94.49	NS
*b*_6_	0.227	1.00	0.044	2.495	0.0547	94.53	NS
*b*_7_	0.520	1.00	0.061	7.027	0.0009	99.91	***
*b*_8_	0.188	1.00	10.285	2.550	0.0512	94.88	NS
*b*_9_	−0.216	1.00	10.285	−2.922	0.0329	96.71	*
*b*_10_	−0.004	1.00	10.285	−0.066	0.9499	5.01	NS
*b*_11_	−0.240	1.00	10.285	−3.246	0.0227	97.73	*

With SE is the standard error and *t*.exp is the value of variables determined by Student's *t*-test.

(***): significant at the level > 99.9% (for 0.0001 < *P* value <0.001).

(**): significant at the levels comprised between 99% and 99.9% (for 0.001 < *P* value <0.01).

(*): significant at the levels comprised between 95% and 99% (for 0.01 < *P* value <0.05).

NS: NonSignificant (Terms were considered NS for *P* value >0.05).

**Table 4:** Three variable CCD design with experimental and predicted values of biosurfactant production by *Bacillus subtilis* SPB1.

Exp N°	Glucose (g/L) *X*_1_ (*x*_1_)	K_2_HPO_4_ (g/L) *X*_2_ (*x*_2_)	Urea (g/L) *X*_3_ (*x*_3_)	Biosurfactant yield (g/L)	
				Experimental value	Predicted value
1	−1 (20)	−1 (1)	−1 (3)	2.900	3.005
2	1 (40)	−1 (1)	−1 (3)	2.400	2.299
3	−1 (20)	1 (2)	−1 (3)	2.600	2.642
4	1 (40)	1 (2)	−1 (3)	2.300	2.337
5	−1 (20)	−1 (1)	+1 (9)	2.800	2.694
6	1 (40)	−1 (1)	+1 (9)	2.100	1.989
7	−1 (20)	1 (2)	+1 (9)	2.400	2.432
8	1 (40)	1 (2)	+1 (9)	2.300	2.127
9	−1.682 (13.18)	0 (1.5)	0 (6)	2.700	2.624
10	+1.682 (46.82)	0 (1.5)	0 (6)	1.600	1.774
11	0 (30)	−1.682 (0.66)	0 (6)	2.900	2.993
12	0 (30)	+1.682 (2.34)	0 (6)	2.800	2.804
13	0 (30)	0 (1.5)	−1.682 (0.95)	2.600	2.518
14	0 (30)	0 (1.5)	+1.682 (11.05)	1.900	2.080
15	0 (30)	0 (1.5)	0 (6)	2.700	2.877
16	0 (30)	0 (1.5)	0 (6)	3.000	2.877
17	0 (30)	0 (1.5)	0 (6)	2.800	2.877
18	0 (30)	0 (1.5)	0 (6)	3.000	2.877
19	0 (30)	0 (1.5)	0 (6)	2.900	2.877

*X* represents the coded level of variables.

*x* represent the actual level of variables.

Figures in parentheses denote actual level of variables.

**Table 5:** Estimated effect, regression coefficient, and corresponding *t* and *P* values for biosurfactant production in central composite design experiments.

Noun	Coefficient	*F* Inflation	Ecart-Type	*t*.exp	Signification
*b*_0_	2.877		0.071537292	40.21	***
*b*_1_	−0.253	1.00	0.043336447	−5.83	***
*b*_2_	−0.056	1.00	0.043336447	−1.03	NS
*b*_3_	−0.130	1.00	0.043336447	−3.00	*
*b*_1-1_	−0.240	1.00	0.043347065	−5.53	***
*b*_2-2_	0.008	1.00	0.043347065	0.18	NS
*b*_3-3_	−0.204	1.00	0.043347065	−4.71	**
*b*_1-2_	0.100	1.00	0.056621757	1.77	NS
*b*_1-3_	0.000	1.00	0.056621757	0.00	NS
*b*_2-3_	0.025	1.00	0.056621757	0.44	NS

(***): significant at the level 99.9%.

(**): significant at the level 99%.

(*): significant at the level 95%.

NS: NonSignificant.

**Table 6:** ANOVA analysis for biosurfactant production in central composite design experiments.

Source of variation	Sum of squares	Degree of freedom	Mean square	*F*-value	Significance
Regression	2.4734	9	0.2748	10.7150	***
Residual	0.2308	9	0.0256		
Lack of fit	0.1628	5	0.0326	1.9157	27.4%
Pure error	0.0680	4	0.0170		

Total	2.7042	18			
